# Race, Ethnicity, and Neighborhood Food Environment Are Associated with Adolescent Sugary Drink Consumption During a 5-Year Community Campaign

**DOI:** 10.1007/s40615-021-01074-9

**Published:** 2021-08-05

**Authors:** Rebecca Boehm, Kristen Cooksey Stowers, Glenn E. Schneider, Marlene B. Schwartz

**Affiliations:** 1 Food and Environment Program, Union of Concerned Scientists, Washington, DC USA; 2 Department of Allied Health Sciences, Rudd Center for Food Policy and Obesity, University of Connecticut, Storrs, CT USA; 3 Horizon Foundation, Columbia, MD USA; 4 Rudd Center for Food Policy and Obesity, Department of Human Development and Family Sciences, University of Connecticut, Hartford, CT USA

**Keywords:** Health equity, Adolescent sugary drink consumption, Neighborhood food retail environment

## Abstract

**Background:**

A multi-level county-wide campaign to reduce sugary drink consumption was associated with significant decreases in retail sales of soda and fruit drinks. The aim of the current study was to examine changes in adolescent beverage consumption during the campaign by race/ethnicity and neighborhood food environment.

**Methods:**

Beverage consumption among adolescents was evaluated at four time points in a repeated cross-sectional survey of a racially and ethnically diverse sample of sixth graders (N = 13,129) from public middle schools in the county. Each school’s surrounding attendance zone (i.e., neighborhoods where students live) was characterized as providing high or low exposure to unhealthy food retail (e.g., convenience stores, fast-food restaurants). Logistic and multiple linear regression models were used to evaluate changes in beverage consumption over time by student race/ethnicity and high versus low unhealthy food exposure.

**Results:**

Over the 5 years, there were significant declines in the overall share of students who reported daily sugary drink consumption (49.4 to 36.9%) and their reported daily calories from these products (220 to 158 calories). However, disparities were observed, with higher levels of consumption among Black and Hispanic youth and among youth living in neighborhoods with more unhealthy food retail. Notably, Black students living in healthier neighborhood food environments reported significant decreases in daily consumption and calories after 5 years, while Black students living in neighborhoods with more convenience stores and fast-food outlets did not.

**Conclusion:**

These findings suggest that both race/ethnicity and neighborhood food environments are important considerations when designing interventions to reduce sugary drink consumption among adolescents.

**Supplementary Information:**

The online version contains supplementary material available at 10.1007/s40615-021-01074-9.

## Introduction

In the USA, children and adolescents dramatically increased their consumption of sugary drinks between 1970 and the early 2000s [1]. This shift is concerning because consumption of sugary drinks increases the risk of diet-related diseases such as obesity, type 2 diabetes, dyslipidemia, and dental caries [2–5]. In response, there have been a range of national efforts to reduce the availability of sugary drinks and discourage their consumption [6–8]. These include policies to remove sugary drinks from schools, child care settings, and workplaces [9–11], and beverage excise taxes [12, 13].

Recent data on sugary drink consumption trends paint a cautiously optimistic picture. For example, recent retail sales data show that the number of beverage calories per person, per day decreased by 5.6% (i.e., from 203.0 to 191.8) between 2014 and 2019 [14]. In addition, earlier evidence from nationally representative, self-reported dietary intake data suggest that there has been a decrease in sugary drink consumption among youth: the proportion of children who consumed a sugary drink on a given day dropped from 79.7% in 2003 to 60.7% in 2014 [15]. Although these findings indicate movement in the right direction, sugary drink consumption remains very high. Furthermore, racial and ethnic disparities persist, with Black and Hispanic youth and adults reporting higher rates of sugary drink consumption than their white peers [15]. These findings highlight the importance of considering race and ethnicity when evaluating changes in sugary drink consumption in general, and in the context of policy and environmental interventions.

In 2013, a county in a mid-Atlantic state launched a multi-year, comprehensive, community-based campaign to specifically reduce sugary drink consumption as part of an overall strategy to address high rates of childhood obesity [16]. A robust evaluation plan using retail sales data and self-report surveys was established prior to the beginning of the campaign. The overall effectiveness of this initiative in reducing sugary drink sales has been demonstrated using a difference-in-differences analysis of retail sales data comparing supermarkets in the target county with a set of matched comparison stores in another state from 2012 (pre-campaign) through the first 3 years of the campaign (2013, 2014, 2015) [17]. In the target county, regular soda sales dropped by 20% and fruit drink sales dropped by 15%, and these decreases were significantly greater than the changes in sales in the comparison stores. A recent follow-up study using the same difference-in-differences analysis approach with weekly beverage retail sales data from 2016 to 2018 found that soda and fruit drink sales have continued to decrease significantly more in the target county stores than in the baseline-matched comparison stores [18].

The observed decrease in sales of soda and fruit drinks in the target county can reasonably be attributed to the campaign; however, from a health equity perspective, it is important to not only examine overall changes in beverage sales, but also assess whether changes in consumption vary across racial and ethnic groups. Kumanyika emphasizes this point in the context of obesity prevention, noting that “the disproportionately high exposure to a variety of obesity-promoting factors in socially disadvantaged communities may limit the effectiveness of interventions that benefit the population at large” [19]. One of these factors is the retail food environment. Indeed, there is a substantial literature that has examined how living in proximity to convenience stores and fast-food restaurants may be linked to lower diet quality and increased risk of obesity [20–22]. For example, a 2017 Baltimore study of a predominantly Black, female sample of 6th and 7th graders showed increased consumption of snacks and desserts among girls living in areas with a higher density of corner stores versus those living in areas with a lower density of corner stores [20]. In a recent systematic review of the research on living near convenience stores, one conclusion was that the literature supports a strong association between access to convenience stores and diet-related behaviors (e.g., snack and sugary drink consumption) among children and adolescents [21]. A second systematic review of the literature on proximity to fast-food restaurants noted mixed findings on several weight-related outcomes; however, most studies found a positive relationship between access to fast-food restaurants and consumption of fast food [22]. Furthermore, findings from a recent survey suggest that racial and ethnic minority populations are more likely to report that convenience stores and fast-food restaurants are the most accessible food retail options in the neighborhoods where they live [23].

Taken together, the literature suggests that there are higher rates of sugary drink consumption among Black and Hispanic youth, and there are higher rates of sugary drink consumption among young people who live near convenience stores and fast-food restaurants. This raises the question: how are “race” and “place” associated with observed disparities in sugary drink consumption? LaViest and colleagues conducted a series of studies in a racially integrated community in Southwest Baltimore over a decade ago and found that many of the racial health disparities observed in national data (e.g., rates of hypertension and diabetes) dissipated when examining a location where Black and white people lived in the same community [24]. They concluded that it is critical to consider the social environment when interpreting observed racial health disparities.

There are three characteristics of the community where the campaign took place that facilitate an examination of how race/ethnicity and neighborhood food environment—independently and in combination—may influence sugary drink consumption among youth. First, the county where the campaign took place is ethnically and racially diverse: 7.3% Hispanic, 50.3% white non-Hispanic, 20.4% Black, 19.3% Asian, and 4.4% two or more/other races [25]. Second, students live and attend school within the same neighborhood. Third, there is variability in the food retail environment across different neighborhoods in the county. Therefore, the aims of the present study are to assess changes in adolescents’ sugary drink consumption over time, and evaluate how their levels of consumption are associated with their race/ethnicity and neighborhood food environment.

## Methods

### Intervention as Environmental Context

Although the current analyses are not designed to test the effectiveness of an intervention, it is important to note that these data were collected in the context of a multi-year, county-wide campaign to reduce sugary drink consumption, especially among school-aged children. In December 2012, a health-focused non-profit foundation joined community partners to launch the campaign. Based on the social-ecological model [26], comprehensive strategies were designed to promote behavior change at interpersonal, organizational, community, and policy levels. The aim was to reach the entire county population, which was approximately 300,000 people in 2012 [25]. A series of successful policy change campaigns: (a) removed student accessible vending machines from all middle schools; (b) set strong nutrition standards for student accessible vending machines in high schools; (c) significantly improved the comprehensiveness and quality of the school district wellness policy; (d) required all childcare facilities to eliminate sugary drinks and serve only healthier beverages; and (e) promoted healthier beverage options in all government-owned vending machines, and in recreation and parks youth programming. The campaign also employed digital marketing ads; cable television commercials; direct mail to households; and sponsored social media posts. An online tool, the Better Beverage Finder, was created to help residents search for healthier beverage options. “Street teams” conducted outreach to market the Better Beverage Finder at pools, parades, sporting activities, and other community events. Healthcare providers were encouraged to counsel their patients on sugary drink consumption and better prevent, diagnose, and treat childhood obesity. Additional details about the components of the campaign are presented in the primary outcome paper on the change in retail sales of beverages during the campaign [17].

### Sample and Survey Administration

The study was approved by the Institutional Review Board at the University of Connecticut. The county where the study took place is a single school district. Surveys were administered to all sixth-grade classes across the middle schools during the school day using an online survey platform. Students were asked about their consumption of sugary drinks as part of a larger set of questions about diet, physical activity, media use, and sleep. The baseline survey was administered in November of the 2012–2013 school year and follow-up surveys were administered for the next four academic years between April and June. The survey took approximately 20 min to complete and classroom teachers were responsible for administration. The completed survey data were accessed and deidentified by the district Research and Program Evaluation office. In school year 2012–2013, students self-reported race/ethnicity, and in 2013–2014 and 2014–2015, students self-reported gender and race/ethnicity. In the 2015–2016 and 2016–2017 surveys, the district linked the survey responses to student records before deidentifying the data and provided demographic variables (e.g., gender and race/ethnicity) in order to reduce participant burden. The school district also provided race/ethnicity data for the full 6th grade class in each school for each year of the survey to allow comparisons between the sample and the population of interest.

### Student Survey Questions

The questions about sugary drink consumption were identical across all years of the survey and were based on items in the California Healthy Eating Active Living Youth Nutrition and Physical Activity Survey and the Boston Youth Survey [27, 28]. Students were asked to report consumption of five types of sugary drinks: regular soda, fruit drinks, sports drinks, energy drinks, and flavored water and teas. Questions included example brands for each drink type and specified not to include “diet” drinks. Students reported consumption frequency as *Never*; *I drink it but not every day*; *1 time per day*; *2–4 times per day*; or *5 or more times per day*. Students then selected the container size they usually consume for each drink type (e.g., glass, can, bottle, pouch, juice box). Daily calories from each target product were calculated by multiplying (a) student consumption frequency; (b) average size in ounces for the selected container type; and (c) average calories per ounce. The nutrition information for each type of drink was obtained from a comprehensive list of 644 sugary drinks commonly marketed to youth at the time of the baseline survey [29]. Appendix Table A1 in the supplementary material lists the calories per ounce by drink and container type. Total daily sugary drink calories were the sum of calories for each drink type per day.

### Assessment of Neighborhood Food Environments

Previous research has defined “food deserts” as residential areas with limited access to affordable, healthy food (often operationalized as distance from a supermarket), and “food swamps” as areas where the availability of fast food and junk food supersedes healthy food options (often operationalized as the ratio of fast food and convenience stores to supermarkets) [30]. However, neither of these metrics are appropriate for the location of this study because this county has excellent access to healthy food options and 100% of the population lives within half a mile of a supermarket [31]. Because our focus is on young adolescents, we quantified the neighborhood food environment based on the prevalence of establishments where youth can independently obtain sugary drinks (i.e., fast-food restaurants, convenience stores, and gas stations with food and beverages) in the neighborhoods where they live and attend school.

We purchased address-level food store data from the National Establishment Time-Series (NETS) Database for 2014 (the midpoint year of the study period). NETS data reflect archival establishment information from Dun and Bradstreet [32]. The micro-level dataset contains each business’ name; address and contact information; years active; and primary industry classification. We obtained data with the North American Classification system codes of 445110 (supermarkets and other grocery stores), 445110 (fast-food restaurants), 445120 (convenience stores), and 447110 (gas stations with food and beverages).

To measure students’ food environments, we utilized the middle school “attendance zones,” which are the neighborhoods surrounding each middle school building where all of the students who attend that school live. We obtained the school attendance zone shape files from the school district and merged them with the food establishment data using ArcGIS Desktop 10.2.2. We defined high exposure to unhealthy food retail zones as school attendance zones where the number of unhealthy food retailers (i.e., fast food, convenience stores, and gas stations) was higher than the in-sample average number of establishments. For our analyses, we constructed a binary variable equal to 1 if the school attendance zone met this criterion and equal to 0 if the number of unhealthy food establishments was below average.

When the study began in 2012, there were 19 middle schools; however, in school year 2015–2016, a new middle school was opened and students were drawn from the three surrounding middle schools. To ensure that we were comparing the same neighborhoods over time, we combined the new school’s attendance zone with the zones of the three surrounding schools to create one combined attendance zone. This did not change the coding of the food environment for any of the schools; each of the three original middle schools were coded as “high exposure” on their own before the new school was built. This designation remained the same for the combined zone of four schools.

### Outcome Measures

The primary sugary drink consumption outcome measures were: share of students reporting daily sugary drink consumption (i.e., ≥ 1 time per day; total and by drink type) and estimated daily calories consumed from sugary drinks (total and by drink type). Students who reported not consuming a beverage were coded as consuming 0 calories from that beverage.

### Data Analysis

Data analyses were completed in Stata/SE 15.0 [33]. First, we assessed the survey response rate. Second, we compared the racial/ethnic distribution of our sample with the racial/ethnic distribution of the full 6th grade population in each school using paired t-tests. Because the method of assessing the race/ethnicity variable changed over time (i.e., self-report during the earlier waves of the survey and drawn from administrative data for the last two waves of the survey), we conducted one set of t-tests with self-report data and a second set with administrative data to examine any shifts in the representativeness of our sample.

Next, we assessed how our independent variable “exposure to unhealthy food retail” was associated with population density (which might explain the higher number of food outlets) and grocery store availability (which would suggest that the number of grocery stores should be included in the assessment of the food environment). We used a t-test to compare the middle school enrollment sizes for high versus low exposure zone schools for 2013–2014, which was the year corresponding to the retail outlet data. A t-test was also used to assess whether the number of grocery stores differed between the high versus low unhealthy retail exposure zones. The mean number of fast-food restaurants and convenience stores/gas stations by exposure zone was also calculated. The proportion of students (overall and by race/ethnicity) living in high exposure zones was calculated and we used chi-square analyses to test whether there were significant differences in the likelihood of specific racial/ethnic groups living in these neighborhoods.

As noted above, we selected two outcome variables: the percent of students reporting daily sugary drink consumption and estimated daily calories consumed from sugary drinks. Logistic regression models were used when the percent of students reporting daily sugary drink consumption was the outcome and linear regression models were used when the number of calories consumed was the outcome. All models included random effects parameters for school-level nesting and robust standard errors. To assess changes over time, the outcomes for each year were compared with 2012–2013 (baseline) levels using unpaired two-sided t-tests with a Bonferroni correction to account for a potential inflation of type 1 error rate from multiple comparisons. Only results significant at *α* = 0.01 are discussed in the text of the “Results” section.

For the first outcome, logistic regression models were used to estimate the percent of students reporting daily sugary drink consumption, comparing baseline levels (2012–2013) to each subsequent year of the survey. This was done for “any sugary drink,” and each of the five types of drinks separately. The analyses that included the full sample were adjusted for student race/ethicity.  We also completed separate models for each of the five racial/ethnic groups.

In the next set of analyses, the students were divided into two groups: those exposed to high versus low levels of unhealthy food retail. We used a t-test to assess whether daily sugary drink consumption was different between high and low exposure zones. Then, logistic regression models were used to estimate the percent of students reporting daily sugary drink consumption, comparing baseline levels (2012–2013) to each subsequent year of the survey. The analyses that included the full sample were adjusted for student race/ethnicity. We also completed separate models for each of the five racial/ethnic groups.

For the second outcome, linear regression models were used to estimate daily calories consumed from sugary drinks, comparing baseline levels (2012–2013) to each subsequent year of the survey. Following the same strategy, this was done for “any sugary drink,” and each of the five types of drinks separately. Again, after the analyses of the full sample (adjusted for student race/ethnicity), separate models were run for each of the five racial/ethnic groups. In the final set of multiple linear regression analyses, the sample was divided into those who live in high versus low exposure zones. We examined estimated daily calories consumed from all sugary drinks and compared baseline levels (2012–2013) to each subsequent year of the survey. This was assessed first for the full sample (adjusted for student race/ethnicity), followed by parallel models for each of the five racial/ethnic groups.

## Results

### Survey Response Rate and Sample Summary Statistics

The survey response rate was 91% in school year 2012–2013, 65% in 2013–2014, 30% 2014–2015, 90% in 2015–2016, and 81% in 2016–2017. In 2014–2015, logistical challenges impeded the survey administration, resulting in a substantially lower response rate; therefore, the data from that year were excluded from all analyses. The sample for the remaining 4 years included 13,129 students between 10 and 13 years old (mean age = 11.7 years, SD = 0.456 years). Table A2 reports the sixth-grade enrollment numbers, sample sizes, and survey response rates, as well as race/ethnicity and gender percentages for each survey year.

Paired t-tests comparing the share of students from each racial/ethnic group in our sample with the full 6th grade population in each school indicate that the racial/ethnic distribution within our sample was not significantly different than the population under study with the exception that we had a higher proportion of multiple race/other students in our sample than were in the 6th grade population (Table A3). The same pattern was identified during the self-report years and the administrative data years, suggesting that the representativeness of our sample was not changed as a consequence of this change in methods.

### Characteristics of Unhealthy Food Retail Zones

There was not a significant difference in the total middle school enrollment numbers in the high exposure versus low exposure zones [*t* = 1.07,p = 0.3952], suggesting that the higher number of unhealthy retail establishments is not attributable to greater population density. In addition, the number of grocery stores located in the high versus low exposure zones was not significantly different [*t* = 1.78,p = 0.0914], supporting the appropriateness of focusing specifically on unhealthy food retail in this community. The average number of fast-food restaurants was 14.2 (SE 4.0) in high exposure zones and 7.3 (SE 1.0) in low exposure zones. The average number of convenience stores and gas stations with food and beverages was 3.8 (SE 0.86) in high exposure zones and 2.4 (SE 0.34) in low exposure zones.

### Race/Ethnicity and Likelihood of Living in High Versus Low Unhealthy Retail Zones

An average of 24.6% of the students lived and attended school in a high unhealthy retail exposure zone (Table A2). Specifically, the proportions of students from each racial/ethnic group living in high exposure zones were 21.6% (Asian); 32.3% (Black); 33.2% (Hispanic); 26.6% (multiple/other race); and 19.8% (white). Although at least two-thirds of students from all racial/ethnic groups lived in low exposure zones, Black, Hispanic, and multiple/other race students were significantly more likely than white and Asian students to live in high exposure zones (*χ*^2^ = 173.55,p < .0001).

### Change Over Time in Daily Sugary Drink Consumption for All Students and by Racial/Ethnic Groups

As presented in Table 1, the percent of students who reported drinking a sugary drink daily declined significantly by 12.5 percentage points over the study period from 49.4% in 2012–2013 to 36.9% in 2016–2017. More specifically, there were significant declines in the percent of students reporting daily consumption of energy drinks (− 2.5 percentage points); fruit drinks (− 7.6 percentage points); regular soda (− 6.9 percentage points); sports drinks (− 7.7 percentage points); and flavored waters and teas (− 5.4 percentage points). Comparing across the different types of sugary drinks, fruit drinks were consistently the most frequently consumed by all students, with nearly 23% of students reporting daily consumption in 2016–2017.

**Table 1:**
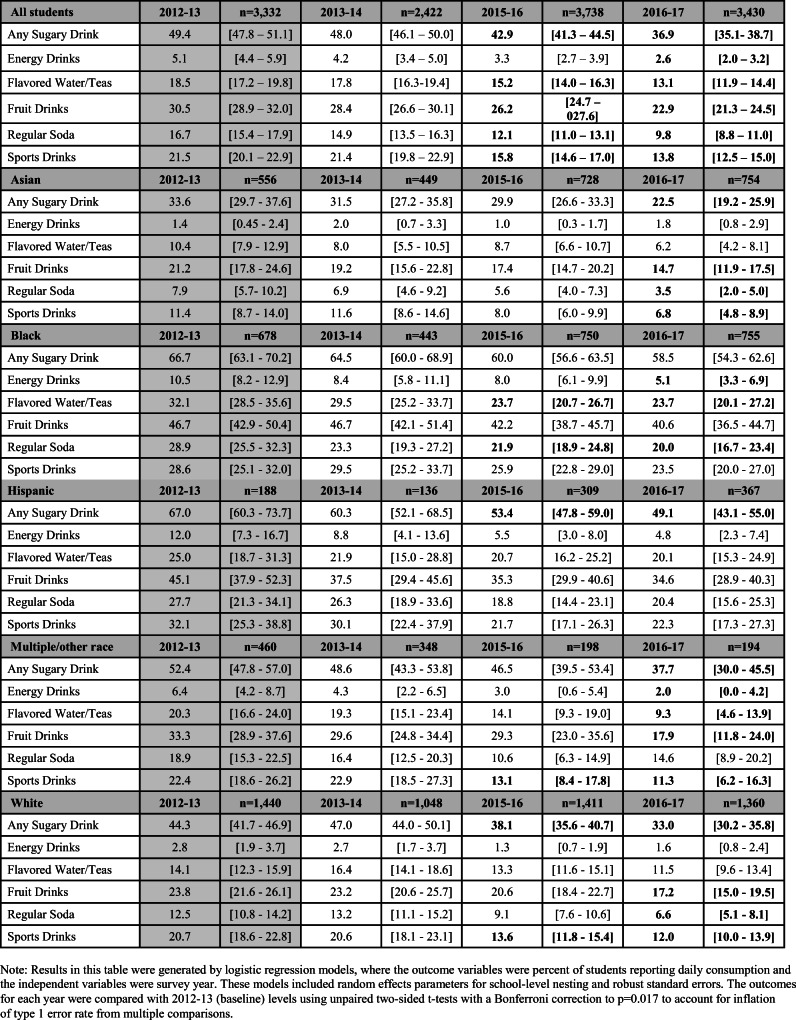
Percent of students reporting daily sugary drink consumption (overall and by type) by race/ethnicity and survey year. Estimated percent of students reported with 95% confidence intervals. Bold indicates a statistically significant difference compared to baseline (2012–2013)

Examining daily consumption by race/ethnicity and beverage type, the results suggest that despite significant decreases in daily sugary drink consumption by 2016–2017 for the sample as a whole, the percent of daily consumers of any sugary drinks remained consistently and considerably higher among Black (58.5%) and Hispanic (49.1%) students than among Asian (22.5%), multiple/other race (37.7%), or white students (33.0%).

### Change Over Time in Daily Sugary Drink Consumption by Exposure to Unhealthy Food Retail and Race/Ethnicity

**Fig. 1:**
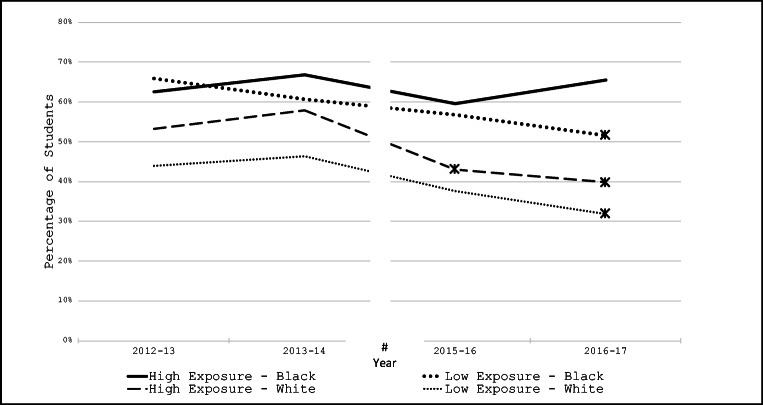
Prevalence of daily sugary drink consumption reported by Black and white students within high versus low unhealthy food exposure zones. # 2014–2015 data were excluded due to small sample size. * Markers indicate significant changes compared to 2012–2013 baseline rates

Overall, the percent of students reporting daily sugary drink consumption throughout the study was significantly higher in high exposure zones versus low exposure zones (mean difference of 7.1 percentage points, t = 6.7, p < 0.0001). Table 2 presents the percent of students reporting daily sugary drink consumption by race/ethnicity and exposure zone. When assessing all of the students together, the percent of daily consumers decreased significantly by 2016–2017 in both high- and low exposure zones, although the percent of daily consumers remained nearly 10 percentage points higher in the high versus low exposure zones (45.3% vs 35.6%). There are notable differences in the percent of daily consumers by both race/ethnicity and exposure to high versus low unhealthy food retail. Asian students consistently reported the lowest rates of daily consumption. For white and Hispanic students, the percent consuming sugary drinks daily in both high and low exposure zones decreased significantly by 2016–2017. In contrast, the percent of Black students consuming sugary drinks daily only dropped significantly among students living in the low exposure zones; the percent of daily consumers in the high exposure zones remained quite high throughout the five years. Multiple/other race students also reported decreases over time, but the change only reached significance for those in the high exposure zones. Figure 1 illustrates the data from Table 2 for white and Black students in the two categories of neighborhood food environments.

**Table 2:**
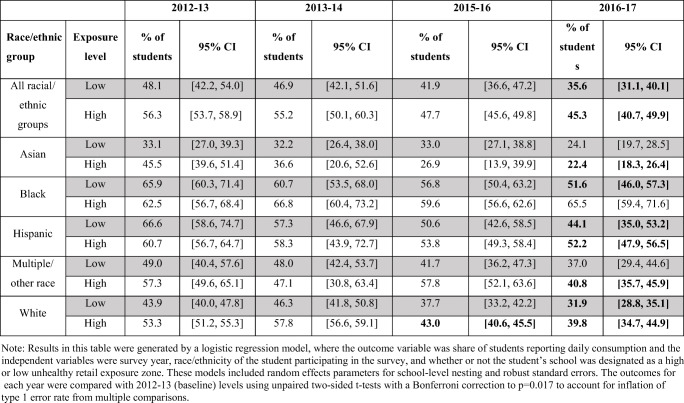
Percent of students consuming sugary drinks daily (any type) by race/ethnicity, survey year, and unhealthy food retail exposure zones (high versus low). Estimated percent of students reported with 95% confidence intervals. Bold indicates a statistically significant difference compared to baseline (2012-2013)

### Change Over Time in Estimated Calories Consumed from Sugary Drinks for All Students and by Racial/Ethnic Groups

For all students, there was a 28.2% decline from 2012–2013 to 2016–2017 in estimated daily calories consumed from all sugary drink types (from 220 to 158 calories) (Table 3). Calories consumed from soda declined quickly and substantially, exhibiting a 32.5% drop between 2012–2013 and 2016–2017. Notably, fruit drinks were consistently the largest source of sugary beverage calories, even though they did decline by 30.2% by 2016–2017. The calories from sports drinks and energy drinks also declined by 32.3% and 46.7% respectively. The calories consumed from flavored water and teas did not decrease significantly over the study period.

**Table 3:**
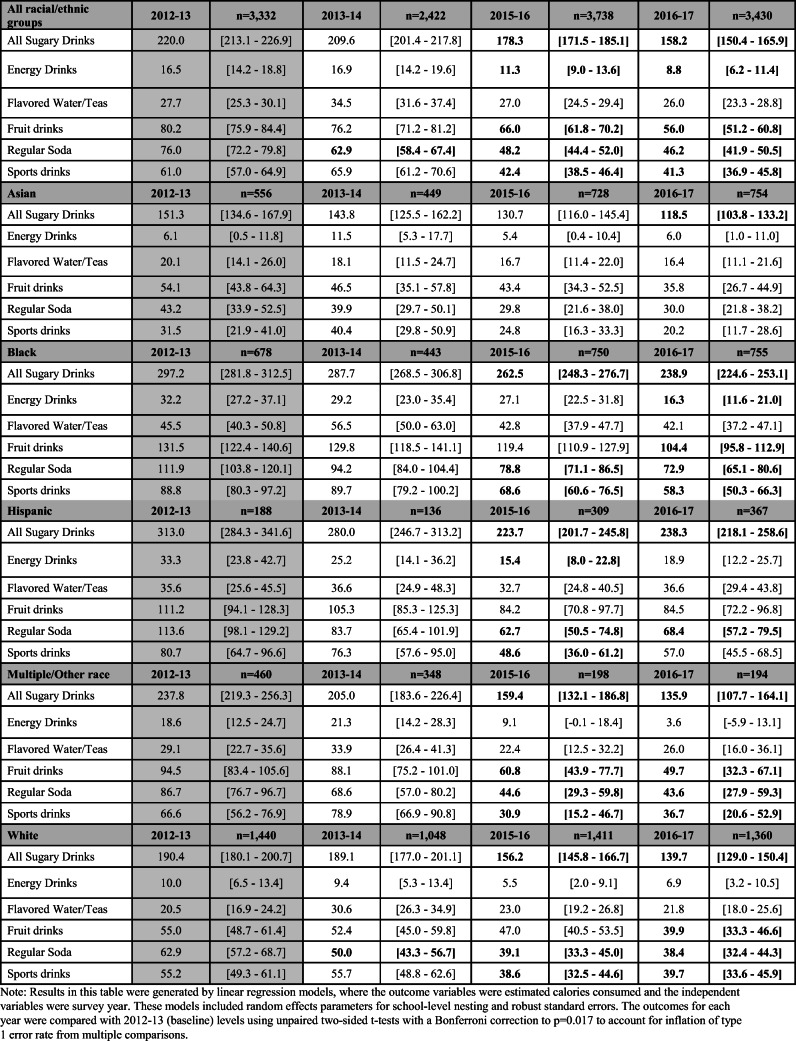
Estimated daily calories consumed from sugary drinks (total and by type) from 2012 to 2017. Estimated daily calories reported with 95% confidence intervals. Bold indicates a statistically significant difference compared to baseline (2012–2013)

As with the proportion of students consuming sugary drinks daily, there were notable differences in the findings by race/ethnicity. Black and Hispanic students reported consuming around 300 calories a day from sugary drinks at baseline, and by 2016–2017 this value had decreased to under 240 calories. White and multiple/other race students started at a lower level and reached under 140 calories a day by 2016–2017. Asian students started at the lowest level of caloric consumption (around 150 calories a day) and reported a significant decline to under 120 calories a day at the end of the study period.

### Change Over Time in Estimated Calories Consumed from Sugary Drinks by Exposure to Unhealthy Food Retail and Race/Ethnicity

Table 4 presents the calories consumed from sugary drinks by race/ethnicity and level of unhealthy food exposure over time. In the full sample, there was a significant decrease in calories consumed between baseline and 2016–2017 for students living in low exposure zones. However, the decrease in calories consumed during the same period of time did not reach significance for the students living in the high exposure zones. This pattern held true for Black and Hispanic students: significant decreases were only achieved for students living in low exposure zones. For Asian, multiple/other race, and white students, significant decreases were reported by students living in both types of neighborhoods.

**Table 4:**
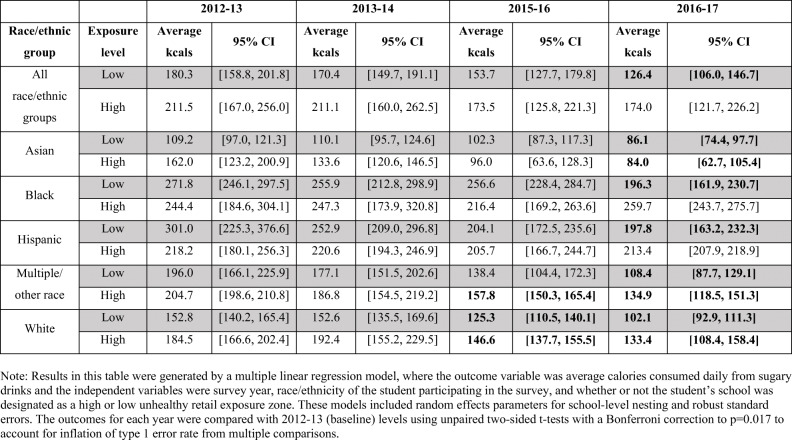
Estimated daily calories consumed from sugary drinks (total) by race/ethnicity, survey year, and high versus low unhealthy food retail exposure zones. Estimated calories reported with 95% confidence intervals. Bold indicates a statistically significant difference compared to baseline (2012 to 2013)

## Discussion

This repeated cross-sectional student survey was conducted during a community campaign to reduce sugary drink consumption. The findings document that between 2012–2013 and 2016–2017 there was a significant decline in self-reported sugary drink consumption among sixth graders attending public schools in the target community. Because national rates of sugary drink consumption have been dropping, and there was not a control group of students unexposed to the campaign, it is not possible to conclude that these changes are due to the campaign. However, this study provided a unique opportunity to carefully assess changes by race/ethnicity and neighborhood food environment.

To put the caloric values reported in this study in context, the Dietary Guidelines for Americans 2020–2025 recommendation is that no more than 7% of calories should come from added sugars, which is 140 calories for a 2000 calorie diet [34]. Although there were declines observed across racial/ethnic groups, many students are still consuming well above this recommended level. In particular, the absolute rates of consumption were consistently higher for Black and Hispanic adolescents than white students, which has been found in previous research [15]. On the other hand, it is notable that sugary drink consumption among Asian students was consistently lower than all of the other groups over time and across both outcome measurements. A strength of the current study is the inclusion of a sizeable Asian population in the sample, and we recommend that future research explore why consumption of sugary drinks is lower among these students.

One reason why Black and Hispanic youth report higher rates of sugary drink consumption may be due to exposure to targeted marketing of these products by beverage companies [35]. There is evidence that Black and Hispanic youth are exposed to more unhealthy food advertising than their white peers on television and through social media [35, 36]. Because of this, public health advocates have argued that efforts to decrease unhealthy food marketing to youth would actually disproportionately benefit children in these populations [37].

A related marketing issue concerns fruit drinks (e.g., Hawaiian Punch, Capri Sun, Sunny-D, and Hi-C), which our study found were the most commonly consumed beverages at all time points. Fruit drinks are heavily marketed as appropriate beverages for children and typically feature pictures of fruit and nutrient content claims such as “all natural,” “no artificial flavors,” and “100% Vitamin-C” on the package [38]. Historically, the calories per ounce for these beverages were comparable to soda; however, companies have reformulated their products in recent years to decrease the amount of added sugar and replace it with non-nutritive (i.e., diet or zero-calorie) sweeteners like sucralose and stevia [38]. Between 2011 and 2019, the percentage of children’s fruit drinks on the market that contained non-nutritive sweeteners increased from 35 to 74% [29, 38]. In addition, a 2019 analysis of children’s drinks found that 65% contained added sugar; 38% contained both sugar and non-nutritive sweeteners; and only 35% contained any juice [38]. There is evidence that parents are confused about the actual contents of these beverages and clearer labeling practices are recommended [39, 40]. Future work is needed to reduce the targeted marketing and consumption of these products by youth, as they contain either added sugar, non-nutritive sweeteners, or both.

A unique feature of the community where this study took place is that the overall racial/ethnic demographic profiles of the students living in the high and low exposure zones were fairly similar. This allowed us to examine different combinations of the food environment and race/ethnicity. The most striking of these was for Black students living in high exposure zones—they were the only subgroup that did reported neither a significant decrease in the percent of daily consumers nor a decrease in the estimated calories consumed daily from sugary drinks between baseline and 2016–2017.  In contrast, Black students living in low exposure zones reported both a decrease in the percent of daily consumers and a decrease in daily calories consumed. This suggests that any forces that were contributing to the decrease in sugary drink consumption overall, and for Black youth in particular, were attenuated by greater exposure to fast-food restaurants, convenience stores, and gas stations that sell food and beverages. These findings support the position that place—in addition to race—is important in our understanding of sugary drink consumption patterns, just as it is for other health disparities [24]. In addition to making specific efforts to engage Black and Hispanic families and youth with counter-marketing that promotes healthier beverages, communities need to consider policies that would decrease the availability and marketing of sugary drinks where children live and go to school.

### Limitations

This study is observational and did not have a matched control group, so we cannot infer a causal  impact of the campaign on sugary drink consumption. There are also well-documented limits to self-reported dietary data, including social desirability bias [41]. The fact that the mode of reporting race and ethnicity changed from student self-report in years 1–3 to administrative data in years 4–5 is also a limitation. Because student-level socioeconomic data (e.g., free/reduced meal status or family income) was not available, we could not control for the students’ financial circumstances. We were also limited to only 1 year of food environment data, so our analysis assumes that the number of unhealthy food retail outlets across the county remained relatively constant over the study period.

### Conclusions

Overall, there were significant decreases in sugary drink consumption reported among a diverse sample of students between the 2012–2013 and 2016–2017 school years. However, racial/ethnic disparities persisted, and exposure to unhealthy retail environments where students live and go to school appeared to reduce the overall positive changes observed. These findings suggest that in order to achieve health equity while reducing sugary drink consumption, interventions and policies are needed to target factors that may be disproportionately affecting Black and Hispanic students, such as reducing the availability and promotion of sugary drinks to youth in fast food and other retail food establishments and prohibiting the marketing of sugary drinks to youth.

## Supplementary Information

ESM 1 (DOCX 27 kb)

## Data Availability

Not applicable.

## References

[1] Della Corte K, Fife J, Gardner A, Murphy BL, Kleis L, Della Corte D, Schwingshackl L, LeCheminant JD, Buyken AE (2021). World trends in sugar-sweetened beverage and dietary sugar intakes in children and adolescents: a systematic review. Nutr Rev.

[2] Vartanian LR, Schwartz MB, Brownell KD (2007). Effects of soft drink consumption on nutrition and health: a systematic review and meta-analysis. Am J Public Health.

[3] Hu FB (2013). Resolved: there is sufficient scientific evidence that decreasing sugar-sweetened beverage consumption will reduce the prevalence of obesity and obesity-related diseases. Obes Rev.

[4] Hu FB, Malik VS (2010). Sugar-sweetened beverages and risk of obesity and type 2 diabetes: epidemiologic evidence. Physiol Behav.

[5] Marshall TA, Curtis AM, Cavanaugh JE, Warren JJ, Levy SM (2021). Beverage intakes and toothbrushing during childhood are associated with caries at age 17 years. J Acad Nutr Diet.

[6] Institute of Medicine Food and Nutrition Board. Accelerating Progress in Obesity Prevention: Solving the Weight of the Nation [Internet]. Washington, D.C.: National Academies Press; 2012. Available from: https://www.nap.edu/catalog/13275/accelerating-progress-in-obesity-prevention-solving-the-weight-of-the. Accessed 21 Apr 2021 24830053

[7] U.S. Department of Health and Human Services and U.S. Department of Agriculture. 2015-2020 Dietary Guidelines for Americans. 8th Edition [Internet]. Washington, D.C.: U.S. Department of Health and Human Services and U.S. Department of Agriculture; 2015 Dec. Available from: https://health.gov/dietaryguidelines/2015/resources/2015-2020_Dietary_Guidelines.pdf. Accessed 21 Apr 2021

[8] Krieger J, Bleich SN, Scarmo S, Ng SW (2021). Sugar-sweetened beverage reduction policies: progress and promise. Annu Rev Public Health.

[9] USDA Food and Nutrition Services. Final rule: National School Lunch Program and School Breakfast Program: nutrition standards for all foods sold in school as required by Healthy Hunger Free Kids Act of 2010 [Internet]. Jun 28, 2013. Available from: https://www.fns.usda.gov/school-meals/fr-072916d. Accessed 21 Apr 2021 23833807

[10] Lee DL, Gurzo K, Nhan LA, Vitale EH, Yoshida S, Hecht K, et al. Status of beverages served to young children in child care after implementation of California policy, 2012–2016. Prev Chronic Dis. 2020;17:190296. 10.5888/pcd17.190296externalicon. 10.5888/pcd17.190296 PMC7207061 32271702

[11] Epel ES, Hartman A, Jacobs LM, Leung C, Cohn MA, Jensen L, et al. Association of a workplace sales ban on sugar-sweetened beverages with employee consumption of sugar-sweetened beverages and health. JAMA Intern Med. 2019;180(1):1–8. 10.1001/jamainternmed.2019.4434. 10.1001/jamainternmed.2019.4434 PMC6820289 31657840

[12] Falbe J, Thompson HR, Becker CM, Rojas N, McCulloch CE, Madsen KA (2016). Impact of the Berkeley excise tax on sugar-sweetened beverage consumption. Am J Public Health.

[13] Lawman HG, Bleich SN, Yan J, Hua SV, Lowery CM, Peterhans A, et al. One-year changes in sugar-sweetened beverage consumers’ purchases following implementation of a beverage tax: a longitudinal quasi-experiment. Am J Clin Nutr. 2020;112(3):644–51. 10.1093/ajcn/nqaa158. 10.1093/ajcn/nqaa158 PMC8491143 32619214

[14] Keybridge Public Policy Economics. 2025 beverage calories initiative: report on 2019 progress toward the National Calorie Goal. September 28, 2020. Available at: https://www.healthiergeneration.org/sites/default/files/documents/20200925/bb4718c7/BCI%202019%20National%20Progress%20Report%20%2809%2025%202020%29%20FINAL%20AHG.pdf. Accessed 21 Apr 2021.

[15] Bleich SN, Vercammen KA, Koma JW, Li Z (2018). Trends in beverage consumption among children and adults, 2003-2014. Obesity (Silver Spring).

[16] Horizon Foundation. Howard County Unsweetened [Internet]. [cited 2021 April 16]. Available from: https://hocounsweetened.org. Accessed 21 Apr 2021.

[17] Schwartz MB, Schneider GE, Choi YY, Li X, Harris J, Andreyeva T, et al. Association of a community campaign for better beverage choices with beverage purchases from supermarkets. JAMA Intern Med. 2017;177(5):666–74. 10.1001/jamainternmed.2016.9650. 10.1001/jamainternmed.2016.9650 PMC5470385 28264077

[18] Schwartz MB, Schneider GE, Xu R., Choi Y-Y, Atoloye A., Highsmith Vernick N., Appel LJ. Retail soda purchases decrease and water purchases increase after six years of a healthy beverage campaign. (Online) American Heart Association EPI/Lifestyle Scientific Sessions. May 2021.

[19] Kumanyika S. Getting to equity in obesity prevention: a new framework. Washington, DC: National Academy of Medicine; 2017. https://nam.edu/wp-content/uploads/2017/01/Getting-to-Equity-in-Obe-sity-Prevention-A-New-Framework.pdf. Accessed 21 Apr 2021.

[20] Hager E, Cockerham A, O’Reilly N, Harrington D, Harding J, Hurley K, et al. Food swamps and food deserts in Baltimore City, MD, USA: associations with dietary behaviours among urban adolescent girls. Public Health Nutr. 2017;20(14):2598–607. 10.1017/S1368980016002123. 10.1017/S1368980016002123 PMC5572508 27652511

[21] Xin J, Zhao L, Wu T, Zhang L, Li Y, Xue H, et al. Association between access to convenience stores and childhood obesity: a systematic review. Obes Rev. 2021;22(Suppl 1):e12908. 10.1111/obr.12908. 10.1111/obr.12908 PMC7988541 31274248

[22] Jia P, Luo M, Li Y, Zheng J-S, Xiao Q, Luo J (2021). Fast-food restaurant, unhealthy eating, and childhood obesity: a systematic review and meta-analysis. Obes Rev.

[23] Cooksey Stowers K, Jiang Q, Atoloye A, Lucan S, Gans K (2020). Racial differences in perceived food swamp and food desert exposure and disparities in self-reported dietary habits. Int J Environ Res Public Health.

[24] LaVeist T, Pollack K, Thorpe R, Fesahazion R, Gaskin D (2011). Place, not race: disparities dissipate in southwest Baltimore when blacks and whites live under similar conditions. Health Aff (Millwood).

[25] United States Census Bureau. Quick facts: Howard County Maryland. Available at: https://www.census.gov/quickfacts/fact/table/howardcountymaryland/PST045219. Accessed April 25, 2021.

[26] Story M, Kaphingst KM, Robinson-O’Brien R, Glanz K (2008). Creating healthy food and eating environments: policy and environmental approaches. Annu Rev Public Health.

[27] Center for Weight and Health. Healthy Eating, Active Living (HEAL) Young Nutrition and Physical Activity Student Survey [Internet]. Berkeley, CA: University of California, Berkeley; 2014. Available from: http://www.farmtoschool.org/resources-main/healthy-eating-active-living-heal-youth-nutrition-and-physical-activity-student-survey. Accessed 21 Apr 2021.

[28] Cradock AL, McHugh A, Mont-Ferguson H, Grant L, Barrett JL, Gortmaker SL, et al. Effect of school district policy change on consumption of sugar-sweetened beverages among high school students, Boston, Massachusetts, 2004-2006. Prev Chronic Dis [Internet]. 2011;8:A74. http://www.ncbi.nlm.nih.gov/pmc/articles/PMC3136975/. Accessed 21 Apr 2021. PMC3136975 21672398

[29] Harris JL, Schwartz MB, LoDolce M, Munsell C, Fleming-Milici F, Elsey J, et al. Sugary Drink FACTS Report [Internet]. New Haven, CT: Rudd Center for Food Policy and Obesity, Yale University; 2011. Available from: http://www.sugarydrinkfacts.org/. Accessed 21 Apr 2021.

[30] Cooksey-Stowers K, Schwartz MB, Brownell KD (2017). Food swamps predict obesity rates better than food deserts in the United States. Int J Environ Res Public Health.

[31] Johns Hopkins Center for a Livable Future. Howard County Maryland Food Systems Profile. 2014. Maryland Food System Map. Available at www.mdfoodsystemmap.org and from authors. Accessed 21 Apr 2021.

[32] Dun and Bradstreet. National Establishment Time-Series database [Internet]. 2012 [cited 2017 Jan 1]. Available from: https://www.dnb.com/. Accessed 21 Apr 2021.

[33] Stata Statistical Software: Release 15. College Station, TX: StataCorp LLC; 2017.

[34] 2015-2020 dietary guidelines [Internet]. [cited 2021 April10]. Available from: https://health.gov/our-work/food-nutrition/2015-2020-dietary-guidelines/guidelines/. Accessed 21 Apr 2021.

[35] Harris JL, Frazier W, Kumanyika S, Ramirez AG. Increasing disparities in unhealthy food advertising targeted to Hispanic and Black youth. January 2019. Available at: http://uconnruddcenter.org/ files/Pdfs/TargetedMarketingReport2019.pdf. Accessed November 30, 2019.

[36] Fleming-Milici F, Harris JL (2020). Adolescents’ engagement with unhealthy food and beverage brands on social media. Appetite..

[37] Kumanyika SK (2019). A Framework for Increasing Equity Impact in Obesity Prevention. Am J Public Health.

[38] Harris JL, Romo Palafox MJ, Choi YY, Kibwana A. Children’s drink FACTS. Sales, nutrition, and marketing of children’s drinks. Rudd Report; 2019. Available from http://sugarydrinkfacts.org/resources/FACTS2019.pdf Accessed April 29, 2021.

[39] Munsell CR, Harris JL, Sarda V, Schwartz MB (2016). Parents’ beliefs about the healthfulness of sugary drink options: opportunities to address misperceptions. Public Health Nutr.

[40] Harris J, Pomeranz J. Misperceptions about added sugar, non-nutritive sweeteners and juice in popular children’s drinks: experimental and cross-sectional study with U.S. parents of young children (1-5 years). Pediatr Obes. 2012:e12791. 10.1111/ijpo.12791. 10.1111/ijpo.12791 33829664

[41] Hebert JR, Clemow L, Pbert L, Ockene IS, Ockene JK (1995). Social desirability bias in dietary self-report may compromise the validity of dietary intake measures. Int J Epidemiol.

