# Supplementary material for: Race, Ethnicity, and Neighborhood Food Environment Are Associated with Adolescent Sugary Drink Consumption During a 5-Year Community Campaign
Source: J Racial Ethn Health Disparities. 2021 Aug 5;9(4):1335–46. doi: 10.1007/s40615-021-01074-9 (PMC9249719; doi:10.1007/s40615-021-01074-9)
Supplement: Supplementary file 1 — (DOCX 27 kb) [file 40615_2021_1074_MOESM1_ESM.docx]

**TITLE:** Race, Ethnicity, and Neighborhood Food Environment are Associated with Adolescent Sugary Drink Consumption During a 5-Year Community Campaign

**JOURNAL:** *Journal of Racial and Ethnic Health Disparities*

**AUTHORS**

Rebecca Boehm^1^, Kristen Cooksey-Stowers^2^, Glenn E. Schneider^3^, and Marlene B. Schwartz^4*^

1. Food and Environment Program, Union of Concerned Scientists, Washington, DC, USA
2. Department of Allied Health Sciences, University of Connecticut, Storrs, CT, USA
3. Horizon Foundation, Columbia, MD, USA
4. Rudd Center for Food Policy and Obesity, University of Connecticut, Hartford, CT, USA

*Corresponding author, [marlene.schwartz@uconn.edu](mailto:marlene.schwartz@uconn.edu)

**SUPPLEMENTAL FILE**

**Table A1. Calories per ounce and container size options by sugary drink type.**

|  | **Calories per ounce** | **Container sizes** |
| --- | --- | --- |
| **Drink type** |  |  |
| Regular soda | 12.5 | Bottle (20 oz.)  Can (12 oz.)  Glass (8 oz.) |
| Fruit drinks | 15.0 | Big glass (12 oz.)  Bottle (16 oz.)  Juice box (6.75 oz.)  Pouch (6.75 oz.)  Small glass (6 oz.) |
| Sports drinks | 8.4 | Glass (8 oz.)  Large bottle (20 oz.)  Small bottle (12 oz.) |
| Energy drinks | 17.5 | Glass (8 oz.)  Large can (16 oz.)  Small can (8.4 oz.) |
| Flavored water/tea | 6.25 | Bottle (20 oz.)  Can (12 oz.)  Glass (8 oz.) |

**Table A2. Survey response rate, sample demographic characteristics, and proportion living in high unhealthy food retail exposure zones**

|  | **Survey Year** | | | |  |
| --- | --- | --- | --- | --- | --- |
|  | **2012-13** | **2013-14** | **2015-16** | **2016-17** | **Total** |
| **Number of students completing survey** | 3,455 | 2,506 | 3,738 | 3,430 | 13,129 |
| **Total number of students enrolled**  **% surveyed** | 3,788  **91%** | 3,855  **65%** | 4,153  **90%** | 4,214  **81%** | 16,010  **82%** |
|  |  |  |  |  |  |
| **Number of students completing survey by racial/ethnic group** | | | | | |
| % White | 43.4 | 42.9 | 41.3 | 39.6 | 40.9 |
| % Black | 20.6 | 18.5 | 22.6 | 22 | 20.3 |
| % Hispanic | 5.6 | 5.6 | 9.0 | 10.7 | 7.4 |
| % Asian | 16.5 | 18.5 | 21.3 | 22 | 19.2 |
| % Multiple/other race | 13.9 | 14.5 | 5.8 | 5.6 | 8.8 |
|  |  |  |  |  |  |
| **% of students completing the survey who reported being female** | -^1^ | 49.4 | 50.2 | 49.3 | 49.6^2^ |
|  |  |  |  |  |  |
| **Percentage of students completing the survey living in high unhealthy food retail exposure zones** | | | | |  |
| % of all students | 24.6 | 20.9 | 27.4 | 24.4 | 24.6 |
| % of White students | 16.8 | 18.7 | 23.3 | 20.4 | 19.8 |
| % of Black students | 36.5 | 21.3 | 36.0 | 31.0 | 32.3 |
| % of Hispanic students | 34.5 | 22.7 | 34.3 | 36.4 | 33.2 |
| % of Asian students | 23.1 | 21.4 | 30.6 | 21.4 | 21.6 |
| % of Multiple/other race | 29.1 | 25.7 | 26.8 | 19.2 | 26.6 |

^1^Student gender was not collected at baseline. ^2^Only includes survey years 2013-14, 2015-16 and 2016-17.

**Table A3. Paired t-tests comparing survey sample and total 6^th^ grade enrollment for racial/ethnic distribution for both assessment methods**

| Survey years | Race/Ethnicity | Survey sample | Total 6^th^ grade enrollment | t-value |
| --- | --- | --- | --- | --- |
| 2012-13  2013-14  (data was self-report) | Asian | .154 | .180 | -1.80 |
|  | Black | .186 | .220 | -2.70 |
|  | Hispanic | .069 | .080 | -0.80 |
|  | Multiple/Other | .119 | .057 | 6.95* |
|  | White | .456 | .461 | -0.20 |
| 2015-16  2016-17  (data obtained from administrative records) |  | | | |
|  | Asian | .220 | .213 | 1.65 |
|  | Black | .222 | .227 | -0.80 |
|  | Hispanic | .092 | .085 | 2.00 |
|  | Multiple/Other | .060 | .043 | 4.45* |
|  | White | .407 | .420 | -1.55 |

*Significance of p=0.05 was adjusted for multiple comparisons with a Bonferroni correction (i.e., p=0.01)
